# 

**Published:** 1904-04-15

**Authors:** 


					﻿THE
DENTAL REGISTER
EDITED BY
NELVILLE S. HOFF, D. D. S.,
ANN ARBOR, MICH.
Vol. LV1II.	APRIL 15, 1904.	No. 4.
PRICE, ONE DOLLAR A YEAR IN ADVANCE.
PUBLISHED MONTHLY BY
SAMUEL A, CROCKER & CO.,
THE OHIO DENTAL AND SURGICAL DEPOT.
3“ to 30 'W'. ötli St., Cincinnati, O.
Entered at the Postoffice at Cincinnati, 0., as second-class mail matter.
				

